# WNT7A-positive dendritic cytonemes control synaptogenesis in cortical neurons

**DOI:** 10.1242/dev.202868

**Published:** 2024-12-09

**Authors:** Thomas M. Piers, Kevin Fang, Seema C. Namboori, Corin Liddle, Sally Rogers, Akshay Bhinge, Richard Killick, Steffen Scholpp

**Affiliations:** ^1^ Living Systems Institute, Department of Biosciences, Faculty of Health and Life Sciences, University of Exeter, EX4 4QD, UK; ^2^ Bioimaging Centre, Department of Biosciences, Faculty of Health and Life Sciences, University of Exeter, Exeter EX4 4QD, UK; ^3^ Department of Old Age Psychiatry, Institute of Psychiatry, Psychology & Neuroscience, King's College London, London SE5 8AF, UK

**Keywords:** WNT7A cytonemes, Synapse formation, IPSC-derived human cortical neurons, Super-resolution imaging, Lattice structured illumination microscopy

## Abstract

Synaptogenesis involves the transformation of dendritic filopodial contacts into stable connections with the exact apposition of synaptic components. Signalling triggered by Wnt/β-catenin and calcium has been postulated to aid this process. However, it is unclear how such a signalling process orchestrates synapse formation to organise the spatial arrangement of synapses along dendrites. We show that WNT7A is loaded on dynamic dendritic filopodia during spine formation in human cortical neurons. WNT7A is present at the tips of the filopodia and the contact sites with dendrites of neighbouring neurons, triggering spatially restricted localisation of the Wnt co-receptor LRP6. Here, we demonstrate that WNT7A at filopodia tips leads to the induction of calcium transients, the clustering of pre- and postsynaptic proteins, and the subsequent transformation into mature spines. Although soluble WNT7A protein can also support synaptogenesis, it fails to provide this degree of spatial information for spine formation and calcium transients, and synaptic markers are induced ectopically along the dendrites. Our data suggest that dendritic filopodia are WNT7A-bearing cytonemes required for focal calcium signalling and initiation of synapse formation, and provide an elegant mechanism for orchestrating the positioning of synapses along dendrites.

## Teaser

**Summary:** Human WNT7A is loaded on dendritic filopodia, triggering localised calcium signalling and synaptic protein clustering to form spines at specific dendrite locations.

## INTRODUCTION

The formation of synaptic connections, or synaptogenesis, is fundamental to development of a functional nervous system and expression of complex sentient behaviours, including learning, emotion, memory formation, and adaption. Altered synapse formation and function have been implicated in the pathophysiology of neurodevelopmental disorders, such as autism spectrum disorder and intellectual disabilities. Synaptic formation requires precise apposition of pre- and postsynaptic components and connections, which, while perceived to be stochastic, rarely form on the first contact, suggesting the involvement of a dynamic, highly regulated process that allows dendritic filopodia to make the correct contact and initiate signalling in a retrograde fashion (Ziv and Smith, 1996).

Substantial progress has been made in identifying the molecular processes involved in synaptic formation. In particular, cell adhesion molecules (CAMs) play a crucial role in synaptogenesis, including L1-CAM and contactin at the presynaptic side and neuroligins and N-cadherin (cadherin 2) at the postsynaptic side (Biederer et al., 2002; Südhof, 2017). However, an improved molecular understanding of how synaptic connections are initiated between neurons is needed. For example, what causes contacts at specific sites to differentiate into synapses but not others? How do pre- and postsynaptic neurons recognise each other and establish a stable connection as an excitatory synapse?

Secreted signalling factors are crucial for promoting synaptogenesis (Packard et al., 2003; Qi et al., 2022). Among these growth factors are the Wnt ligands, which play a prominent role in orchestrating adult neural development and neuronal plasticity (McLeod and Salinas, 2018). Strong evidence suggests that Wnt proteins influence cellular behaviour at the tissue level and activate localised signalling at specific sites on the plasma membrane that regulate dynamic cytoskeletal changes (Lai et al., 2009; Routledge and Scholpp, 2019). More specifically, the Wnt/β-catenin signalling pathway triggered by Wnt7a has been shown in mice to activate an essential cascade required for promoting the transformation of thin spines into a mushroom-like morphology, which can be seen as an indicator for initiating and forming synapses (Ciani et al., 2011; Hall et al., 2000). However, precisely how Wnt7a signalling induces and maintains synapses remains poorly understood.

Historically, the involvement of Wnt signalling in synaptogenesis has focussed on a diffusion hypothesis, whereby Wnt ligands are released from postsynaptic cells acting in a retrograde fashion to recruit presynaptic proteins, followed by bidirectional Wnt signalling and subsequent dendritic spine morphogenesis (Dickins and Salinas, 2013). However, two controversial issues with this model are the unfocussed nature of diffusion, which contrasts with the highly spatial organisation of the spines along neurites, and the Wnt ligands' hydrophobicity, which prevents free diffusion of the ligand. Therefore, how these ligands are targeted to specific regions on receiving neurons and how these lipophilic growth factors are trafficked intercellularly through the aqueous extracellular space is unclear.

Recently, we and others have identified signalling filopodia, also known as cytonemes, as an essential transport and signalling mechanism. Cytonemes can bring lipophilic Wnt proteins to a target cell without the need for an extracellular release from the donor cell (Zhang and Scholpp, 2019). Wnt ligands such as Wnt3a, Wnt8a, Wnt5a and Wnt5b have been shown to be transported by cytonemes in *Drosophila*, zebrafish and human tissue culture cells (Huang and Kornberg, 2016; Stanganello et al., 2015; Mattes et al., 2018; Routledge et al., 2022; Rogers et al., 2023; Zhang et al., 2024; Kornberg and Roy, 2014). Furthermore, Wnt signalling, transmitted via cytonemes, co-activates calcium signalling (Junyent et al., 2020). Finally, recent studies in *Drosophila* have shown that these cytonemes have structural similarities with neuronal synapses and that signalling facilitates glutamatergic signalling in this context (Huang et al., 2019). Collectively, these findings have begun to alter our mechanistic understanding of the role of Wnt signal presentation in synaptogenesis. Here, we propose that dendritic filopodia are a form of Wnt signalling cytonemes and that their contact sites are the precursors to synaptic formation.

Using super-resolution lattice structured illumination microscopy (L-SIM) microscopy and mature human induced pluripotent stem cell (iPSC)-derived cortical neuron cultures, we show that WNT7A-positive filopodia make contact with adjacent neurons, leading to local clustering of the Wnt co-receptor LRP6 along dendrites. Concomitantly, we observe focal calcium signalling at these contacts. Following local Wnt/β-catenin and calcium signalling, we find clustering of the postsynaptic density marker 95 (PSD95; DLG4) on the dendritic filopodia membrane and bassoon (BSN) on the opposing, pre-synaptic side followed by the morphological transformation from dendritic filopodia into mushroom-type spines. Tethering WNT7A to the dendritic filopodia plasma membrane is required in this process to allow precise spatial organisation in synaptogenesis. Consistently, the inactivation of membrane-tethered Wnts inhibits synapse formation. Extracellularly provided WNT7A protein can support synaptogenesis but lacks this spatial information for localised contact formation. Thus, we conclude that dendritic filopodia resemble many features of Wnt-bearing cytonemes and that the transport of membrane-tethered WNT7A by dendritic cytonemes is a crucial prerequisite to the formation of excitatory synapse formation at a precise location.

## RESULTS

### Most dendritic protrusions harbour WNT7A and induce clustering of LRP6

**Fig. 1.**
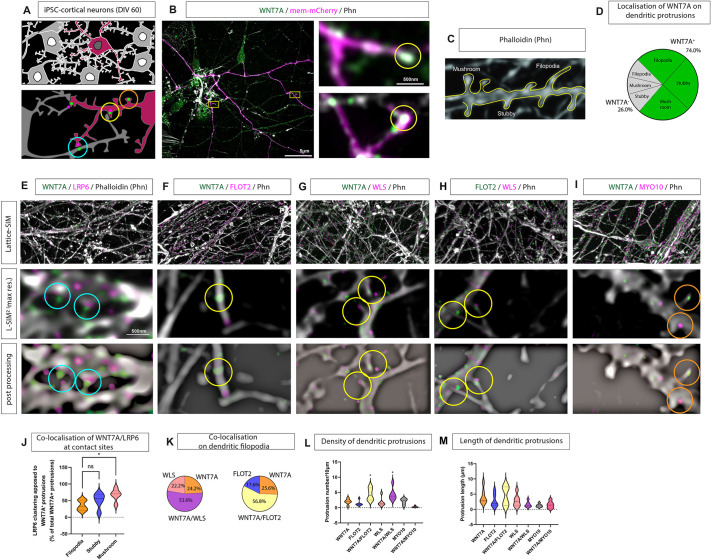
**Characterisation of dendritic filopodia as WNT7A-carrying signalling filopodia.** (A) Schematic of iPSC-derived cortical neuron cultures at 60 days *in vitro* (DIV 60), transfected with membrane marker and co-stained with antibodies to observe localisation of WNT7A, LRP6, and classical filopodia/cytoneme markers. Blue circles denote areas of opposing localisation of proteins across membranes, yellow circles denote proper protein colocalisation, and orange circles no colocalisation. (B) Super-resolution imaging of iPSC-derived cortical neurons stained with anti-WNT7A antibody shows localisation of the protein to dendritic protrusions. Boxed areas are shown at higher magnification on the right. (C,D) Quantification (D) of dendritic protrusion types (filopodia, stubby, mushroom; C) found that nearly 75% of total protrusions were WNT7A positive with no difference in the distribution of WNT7A protein across the class of protrusion. (E) Super-resolution microscopy of neurons co-stained with antibodies against WNT7A and LRP6 shows localisation of WNT7A on dendritic protrusions, whereas LRP6 generally localises at opposing membranes (E, blue circles). WNT7A-positive protrusions also harbour proteins associated with Wnt-signalling filopodia, such as flotillin 2 (FLOT2; F, yellow circles) and Wntless/evenness interrupted (WLS; G, yellow circles), with over 50% of WNT7A-positive protrusions colocalising with these cytoneme markers. Conversely, the Wnt-signalling filopodia marker Myo-10 localises to a WNT7A-negative subset of protrusions (I, orange circles). FLOT2 and WLS colocalise on the same protrusions (H, yellow circles). (J) Quantification of LRP6 and WNT7A co-clustering at apposing membranes identified a significant increase in co-clustering of WNT7A-positive mushroom-shaped protrusions and LRP6 compared to co-clustering at filopodia contacts. (K) Quantification of protrusion number based on cytoneme markers found significantly more WNT7A/FLOT2-positive and WNT7A/WLS-positive protrusions compared to WNT7A-only positive protrusions, and 74% of all protrusion analysed were WNT7A positive. (L,M) Quantification of protrusion density (L) and length (M) found no difference based on the colocalisation of WNT7A with cytoneme protein markers. Statistical significance was addressed using one-way ANOVA with Dunnett's multiple comparison test to compare relevant controls within groups. *P<0.05. ns, not significant.

Wnt/β-catenin signalling is an essential signalling network in the development of the nervous system, with WNT7A specifically being suggested to aid the formation of synaptic connections (Dickins and Salinas, 2013). However, the precise site of WNT7A activity during synaptogenesis is unclear. To map the location of WNT7A during synaptogenesis, we established iPSC-derived cortical neuron cultures (iNeurons; Fig. S1A-G). During the differentiation process, we found that after neural induction neural progenitors expressed PAX6, KI67 (MKI67), vimentin, and TBR1 (Fig. S1A-C). After ∼40 days *in vitro*, we observed localisation of the pre- and postsynaptic markers bassoon and PSD95 (Fig. S1D,E), and these iNeurons formed a complex network of neurites after day 60 (Fig. S1E-G). Using these iNeurons, we analysed dendritic protrusions after 60 days *in vitro* (Fig. 1A). Using Lattice SIM-squared (L-SIM2) with a lateral resolution below 100 nm (Löschberger et al., 2021 preprint), we found a signal for the Wnt ligand WNT7A present on 74% of all protrusions accumulated at filopodia contact sites (Fig. 1B-D, antibody controls; Fig. S2A-F). Similarly, we observed WNT7A at the tips of filopodia in differentiated neurons in an SH-SY5Y-derived cell culture system (Fig. S1H,I). The number of WNT7A-positive protrusions increased with maturity at neurites of iNeurons. These protrusions were positive for the Wnt/β-catenin pathway co-receptor LRP6 suggesting trans-activation of this pathway from the postsynaptic neuron to the pre-synaptic side (Fig. 1E,J, blue circles). Intracellular Wnt trafficking requires specific, chaperone-like proteins (Bischoff et al., 2013; Routledge et al., 2022). On the same filopodia, we found colocalisation of WNT7A with the Wnt ligand transporter, Wntless/evenness interrupted (WLS), and the membrane-bound scaffolding protein and cytoneme marker flotillin 2 (FLOT2; Fig. 1F-H). The number of WNT7A/WLS/FLOT2-positive protrusions was higher than protrusions carrying individual components (Fig. 1K), suggesting co-dependency. Further supporting this notion, we also observed an increased density of WNT7A/WLS- and WNT7A/FLOT2-positive filopodia (Fig. 1L). Finally, the longest dendritic filopodia carried both WNT7A and FLOT2 (Fig. 1M). As these dendritic filopodia were loaded with WNT7A protein and clustered LRP6 at the contact site, we define them as WNT7A cytonemes. Interestingly, we could not detect the actin motor myosin 10 (MYO10), which regulates the formation and elongation of filopodia (Berg and Cheney, 2002) on WNT7A-positive dendritic filopodia (Fig. 1I), suggesting a difference from the Wnt8a cytonemes observed in zebrafish gastrulation (Stanganello et al., 2015) or WNT3 cytonemes in human gastric cancer (Routledge et al., 2022).

**Fig. 2.**
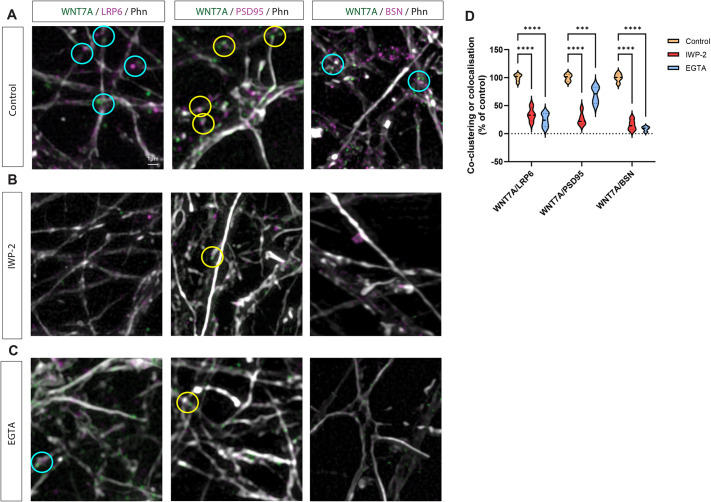
**Lipidation of Wnt and calcium are required for colocalisation of WNT7A and synaptic markers.** (A-C) Antibody staining for WNT7A and LRP6, WNT7A and PSD95, or WNT7A and bassoon (BSN) in iPSC-derived cortical neurons in the absence (A) or presence of the porcupine inhibitor IWP-2 (B), or the calcium chelator EGTA (C). Phn, phalloidin. (D) Quantification of the co-clustering or colocalisation of WNT7A/LRP6, WNT7A/PSD95, and WNT7A/BSN shows dependence on intracellular Wnt trafficking and calcium signalling. Statistical significance was addressed using one-way ANOVA with Dunnett's multiple comparison test to compare relevant controls within groups. ***P<0.005; ****P<0.001.

Next, we tested whether WNT7A localised on dendritic filopodia tips can influence the appearance of pre- and postsynaptic markers. To this end, we co-stained the neuronal cultures with WNT7A and LRP6, and used bassoon to identify the pre-synaptic side and PSD95 as a postsynaptic marker. We observed a strong colocalisation of these markers, specifically on dendritic filopodia contact sites (Fig. 2A,D). Next, we tested whether functional WNT7A is required for the observed clustering. We inactivated WNT7A function by inhibiting porcupine O-acyltransferase-mediated palmitoleoylation of Wnt proteins by treating the cultures with IWP-2. Indeed, we found that the clustering of LRP6 in neurites apposing WNT7A-positive protrusions was dependent on lipidated, functional Wnt ligand (Fig. 2B,D). Finally, we investigated whether clustering of these synaptic markers required calcium signalling. Similarly, after the inhibition of calcium signalling by EGTA, we did not observe colocalisation of WNT7A with LRP6, BSN, or PSD95 (Fig. 2C,D). These data suggest that clustering the pre- and postsynaptic markers at dendritic filopodia contact sites are co-dependent on active Wnt and calcium signalling.

### Membrane-tethered WNT7A-GFP can cluster Wnt and synaptic components

**Fig. 3.**
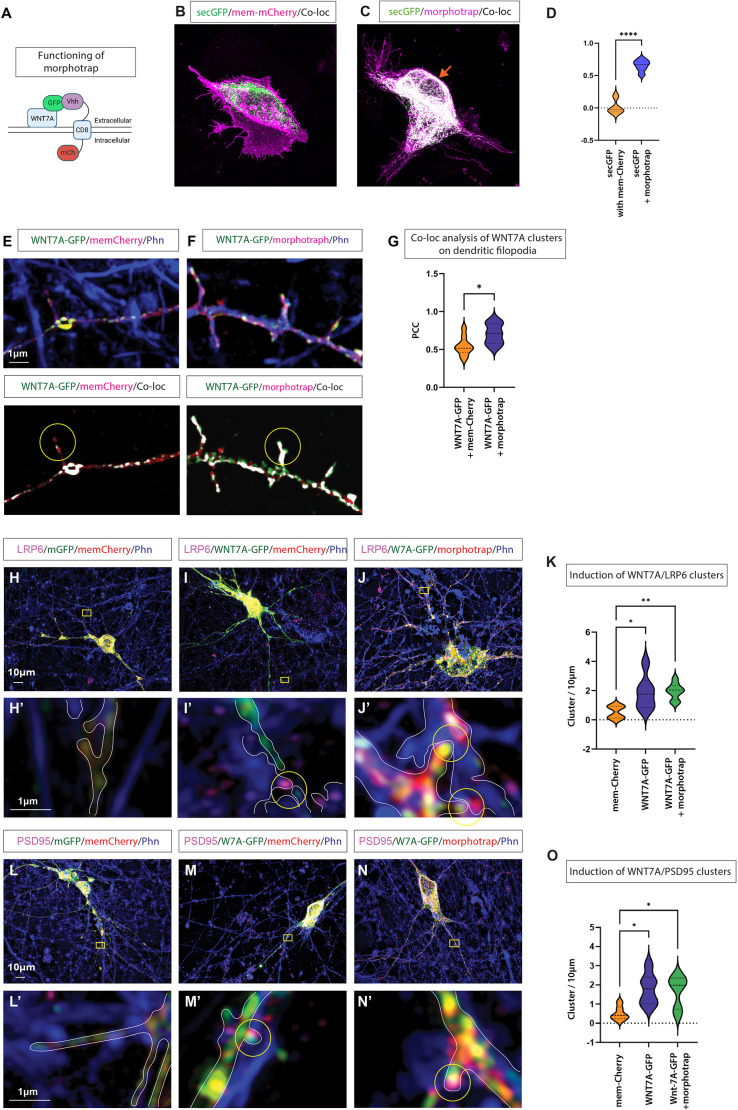
**Membrane-tethered WNT7A-GFP can cluster Wnt and synaptic components.** (A) Schematic of the tethering experiment using double transfection of WNT7A-GFP and the morphotrap nanobody construct, variable heavy domain of the heavy chain (Vhh)-CD8-mCherry, which binds GFP-tagged proteins. (B,C) AGS cells transfected with soluble-GFP (secGFP) and mem-mCherry (B) and AGS cells transfected with secGFP and morphotrap (C). Orange arrow indicates membrane localisation of secGFP. (D) Quantification identifies a significant colocalisation between the normally secreted secGFP and the morphotrap at the plasma membrane. An unpaired, one-tailed Student's *t*-test (****P<0.001). (E) The colocalisation of mem-mCherry and WNT7A-GFP on protrusions is shown in white. (F,G) Significantly higher WNT7A-GFP colocalisation on filopodia is observed when co-transfected with morphotrap (F), quantified by Pearson's correlation coefficient (PCC; G). Unpaired, one-tailed Student's *t*-test (*P<0.05). (H,H′) Super-resolution imaging of iPSC-derived cortical neurons transfected with mem-GFP and mem-mCherry, followed by post-staining for LRP6 and the actin cytoskeleton (phalloidin, Phn). (I,I′) Transfection with WNT7A-GFP and mem-mCherry, followed by post-staining for LRP6 and the actin cytoskeleton identified WNT7A-GFP-positive protrusions that cluster LRP6 in apposed cells (I′, yellow circle). (J,J′) Transfection with WNT7A-GFP and morphotrap, followed by post-staining for LRP6 and cytoskeleton, identified protrusions harbouring membrane-tethered WNT7A-GFP that cluster LRP6 in apposed cells (J′, yellow circles). (K) Quantification of co-clustering of LRP6 with WNT7A-GFP identified a significant increase in both the morphotrap and mem-mCherry conditions compared to mem-Ch alone. Unpaired, one-tailed Student's *t*-test (*P<0.05, **P<0.01). (L,L′) Transfection with mem-GFP and mem-mCherry, followed by post-staining for PSD95 and the actin cytoskeleton (Phn). (M,M′) Transfection with WNT7A-GFP and mem-mCherry, followed by post-staining for PSD95 and actin, revealed colocalisation on dendritic protrusions (M′, yellow circle). (N,N′) WNT7A-GFP co-transfected with morphotrap and colocalised with PSD95 on dendritic protrusions (N′, yellow circle). (O) Quantification of the colocalisation of PSD95 with WNT7A-GFP also identified a significant increase in both the morphotrap and mem-mCherry conditions compared to mem-Ch alone. Experiments were performed on three independent biological replicates. A minimum of three transfected neurons were quantified per condition. Statistical significance was addressed using one-way ANOVA with Dunnett's post-hoc test for multiple comparisons, comparing groups to the mem-mCherry/mem-GFP control group. *P<0.05. In H-J,L-N, boxed areas indicate the area shown at higher magnification in H′-J′,L′-N′.

Identifying localised WNT7A on dendritic filopodia and clustering of LRP6 on the opposing neurite suggest a precise, focal signalling event from one neuron to a neighbouring neuron. However, Wnts have also been considered to be secreted proteins that diffuse extracellularly in some contexts. Therefore, we established a platform to test whether membrane-tethered Wnt proteins can cluster Wnt receptors and synaptic markers. Consequently, we used the membrane-anchoring morphotrap, the membrane-spanning GFP nanobody Vhh-CD8-mCh (Harmansa et al., 2015). Morphotrap tethers GFP-tagged proteins to the membrane through the single-pass CD8 transmembrane domain (Fig. 3A-C, orange arrow) and indeed morphotrap can significantly increase the localisation of secreted GFP (secGFP) to the plasma membrane (Fig. 3D). To analyse whether WNT7A can signal *in trans* without diffusion into the extracellular space, we co-expressed a GFP-tagged active WNT7A (WNT7A-GFP) together with morphotrap in cortical neurons and found a stronger membrane association of WNT7A-GFP with the mCherry-tagged morphotrap, compared to the colocalisation of WNT7A-GFP with mem-mCherry on dendritic filopodia (Fig. 3E-G). These findings suggest that morphotrap efficiently tethers the WNT7A-GFP protein to the membrane. Next, we wanted to test whether membrane-tethered WNT7A can activate Wnt signalling at cytoneme-contact sites *in trans* in iNeurons, similar to the non-tethered form. Therefore, we post-stained cultures for endogenous LRP6 24 h after co-transfecting WNT7A-GFP with either mem-mCherry or morphotrap. We found a significant induction of LRP6 clustering at the opposing membrane when WNT7A-GFP was overexpressed compared to control transfected iNeurons (Fig. 3H-I′). Next, we used morphotrap to tether WNT7A-GFP to the membrane and found that the clustering of LRP6 *in trans* did not differ (Fig. 3J-K), suggesting that membrane-associated WNT7A signals can signal *in trans*. Next, we tested for the ability of membrane-tethered WNT7A signalling to promote synaptic marker clustering, as suggested previously (Fig. 2). Therefore, we stained our iNeuron cultures for PSD95. We observed PSD95 clusters on WNT7A-positive protrusions compared to control neurons (Fig. 3L-M′). Similarly, the formation of PSD95 clusters did not change when morphotrap kept WNT7A at the membrane (Fig. 3N-O). Our results suggest that membrane-tethered WNT7A can activate Wnt signalling *in trans* and promote the clustering of synaptic markers. Therefore, we propose that a diffusible form of Wnt protein is not required in this process.

### memNotum inactivates Wnts at the plasma membrane

**Fig. 4.**
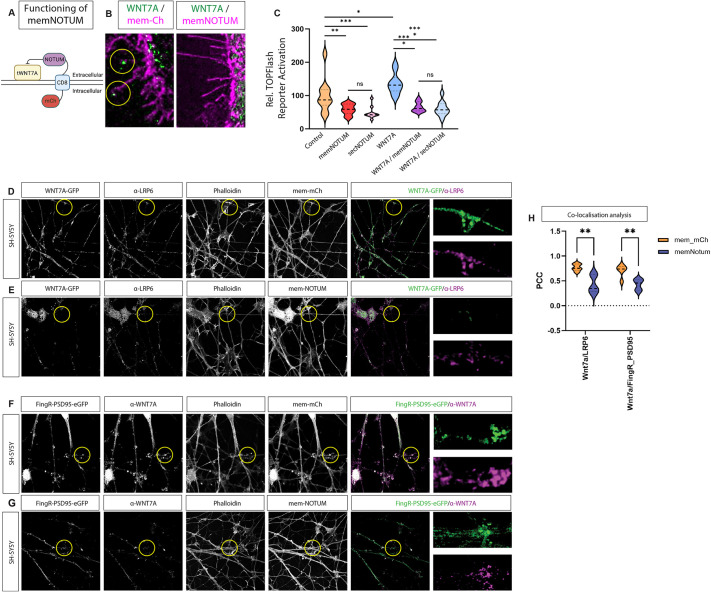
**Calcium transients on dendritic protrusions depend on membrane-associated Wnts.** (A) Schematic of the generated mCherry-tagged membrane-tethered NOTUM construct (memNOTUM). mCh, mCherry. (B) Co-transfection of the membrane marker mem-mCherry and WNT7A-GFP shows localisation of WNT7A-GFP to cytoneme tips (yellow circles), whereas co-transfection of memNotum and WNT7A-GFP reduces WNT7A-GFP-positive filopodia. (C) Quantification of mCherry nuclear expression in the receiving cell population identified the ability of memNotum to significantly reduce Wnt-mediated paracrine signalling to a similar level as secreted NOTUM (transfection of full-length untethered human NOTUM). Statistical significance was addressed using one-way ANOVA with Dunnett's multiple comparison test to compare relevant controls within groups and an unpaired, one-tailed Student's *t*-test to compare specific combinations. *P<0.05; **P<0.01; ***P<0.005. ns, not significant. Experiments were performed in biological triplicate, with three fields per group analysed. (D,E) SH-SY5Y differentiated neurons transfected with WNT7A-GFP and mem-mCherry followed by post-staining with anti-LRP6 show strong colocalisation (D), which is reduced when neurons are transfected with memNotum (E). (F-H) SH-SY5Y neurons transfected with FingR-PSD95 and memNotum and post-stained with anti-WNT7A also show reduced colocalisation of the PSD95/WNT7A proteins compared to the mem-mCherry control (F,G) signal. Quantification is shown in H. signal. Two-way ANOVA with Bonferroni's post-hoc test for multiple comparisons was performed for statistical comparisons between each group. **P<0.01.

Given that we have identified functional WNT7A-bearing cytonemes, we postulated that the combination of localised Wnt and calcium signalling might provide a mechanism to guide dendritic filopodia to precise focal locations on the pre-synaptic neurite. To test for the requirement of membrane-associated WNT7A in this process, we developed a tool to inactivate Wnt proteins specifically at the plasma membrane. Therefore, we generated a membrane-bound Wnt scissor, a Notum-CD8-mCherry (memNOTUM). NOTUM is a carboxylesterase that inactivates Wnt proteins by removing the essential palmitoleate moiety from Wnt proteins (Kakugawa et al., 2015). The palmitoleate is necessary for membrane localisation as it interacts with the Wnt chaperone WLS and the surface glypicans, and for signalling, as it is required for the interaction with the FZD receptors. We fused the extracellular carboxylesterase with a single-pass CD8 domain and an intracellular mCherry (Fig. 4A). Overexpression showed robust localisation at the plasma membrane, specifically on filopodia (Fig. S3A,B). To characterise this tool further, we co-expressed WNT7A-GFP in AGS cells. We found a prominent localisation of the protein at filopodia tips (Fig. 4B, Fig. S3C). Next, we co-expressed the membrane-bound Wnt scissor, demonstrating a selective removal of WNT7A-GFP from the plasma membrane, including cytonemes (Fig. 4B, Fig. S3D). In contrast, intracellular compartments were less affected. As NOTUM cleaves the palmitoleate moiety of Wnts, we hypothesised that Wnts expressed in the same cell as memNOTUM do not only lose their membrane localisation but also become non-functional. To test for functionality, we transfected WNT7A with or without memNOTUM and co-cultured these cells with cells expressing the Wnt TOPFlash reporter TCF7-NLS-mCherry (Fig. S3E-H). We found that memNOTUM has a similar effect on paracrine Wnt signalling compared to the secreted wild-type (WT) form of NOTUM (Fig. 4C). Next, we co-transfected the memNotum source cells with WNT7A, which also significantly blocked paracrine WNT7A activation, compared to WNT7A only-expressing cells. To compare it to the secreted form of NOTUM, we co-transfected WNT7A together with the WT NOTUM. Also, in this context, we did not observe a difference in paracrine Wnt activation between memNOTUM and secreted WT NOTUM. These findings suggest that membrane-bound NOTUM can effectively block Wnt activation because WNT7A is predominantly membrane associated.

Next, we tested the requirement of membrane-tethered WNT7A in a neuronal context. Therefore, we used neurons differentiated from the neuroblastoma cell line SH-SY5Y. We wanted to determine whether memNOTUM can also inhibit WNT7A and, thus, also the clustering of LRP6. Therefore, we transfected SH-SY5Y neurons with WNT7A-GFP and found endogenous LRP6 clustering at the sites where we observed WNT7A-GFP (Fig. 4D). Consistently, the co-expression of WNT7A-GFP with memNOTUM reduced WNT7A-GFP from the membrane of the dendrites compared to the control neurons (Fig. 4E). Thus, we found a significant reduction of WNT7A-GFP/LRP6 clusters (Fig. 4H). Then, we tested whether membrane-tethered WNT7A is also required for clustering of the postsynaptic marker PSD95. We transfected SH-SY5Y neurons with fibronectin intrabodies generated by an mRNA display (FingR) construct that tags endogenous levels of the postsynaptic scaffolding protein PSD95 with GFP (FingR-PSD95; Gross et al., 2013). Then, we stained these neurons for endogenous WNT7A. We found that WNT7A can cluster PSD95 in dendrites of SH-SY5Y neurons (Fig. 4F). Next, we co-expressed memNOTUM and found a significant reduction of the WNT7A and, consequently, PSD95 clusters on the dendrites (Fig. 4G,H). These data suggest that membrane-tethered WNT7A is necessary and sufficient to cluster LRP6 and PSD95 on dendrites of SH-SY5Y neurons.

### Calcium transients on dendritic protrusions depend on membrane-associated Wnts

**Fig. 5.**
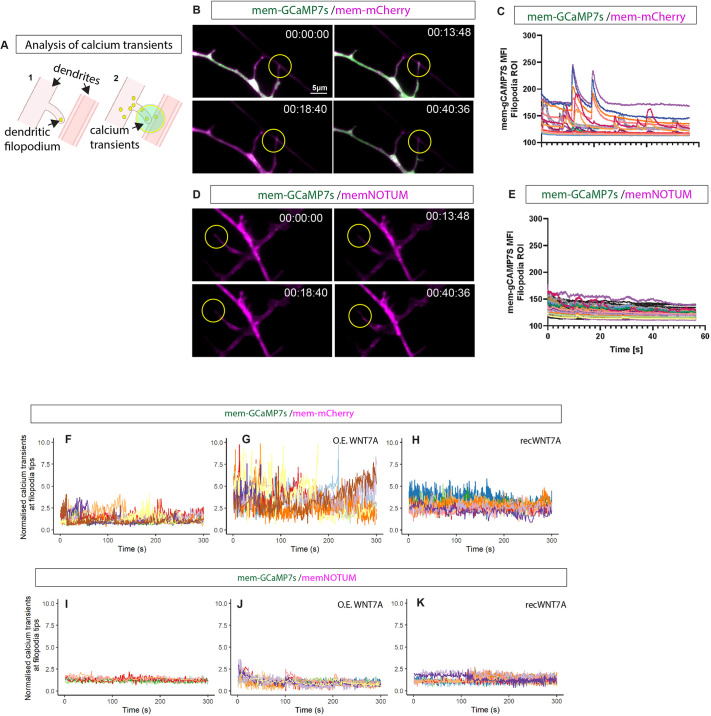
**Effect of Wnt ‘scissor’ on calcium transient induction at filopodia contact sites.** (A) Schematic of calcium transient analysis from live-cell imaging studies performed on the Elyra7 L-SIM2 system, with (1) showing the analysis of filopodia contacts to near neighbouring neurites leading to (2) calcium transients measured in a 2.5 μm radius of the contact point. (B,C) Representative frames from live imaging of iPSC-derived cortical neurons transfected with either mem-mCherry and mem-gCaMP7s performing continuous calcium imaging (1-min bursts) in HILO mode to obtain 10 μm optical sections (min:s:ms) (B) shows classic calcium transient signals. Quantification is shown in C. (D,E) When cells were transfected with membrane-tethered NOTUM (memNotum) and mem-gCaMP7S (D) calcium transients were lost (E). (F-H) Representative traces from calcium transients occurring specifically on filopodia identified increases in transient amplitudes when either overexpressing WNT7A (G) or when exogenously applying WNT7A (H). (I-K) A reduction in calcium transient amplitude and frequency were observed when cells were co-transfected with membrane-bound NOTUM (memNotum) at basal levels, but also when either transfected with WNT7A (O.E. WNT7A; J) or after exogenous application of recombinant WNT7A (K). Live calcium imaging experiments were performed in three independent batches, with at least two independent transfections per condition. Filopodial calcium transients were analysed on at least eight filopodia/condition/batch. The dendritic calcium transients and protrusion spacing were analysed on a subset of this dataset (nine fields from three independent experiments).

Having established that membrane-tethered WNT7A is essential for synaptic marker clustering in SH-SY5Y neurons, we wanted to test the requirement of Wnts transported on dendritic cytonemes for synaptogenesis. As synapses develop and become functional, they exhibit activity-dependent calcium transients in response to synaptic activity (Lohmann and Bonhoeffer, 2008). Therefore, as a measure of synapse maturation, we first measured calcium signalling during synapse maturation, specifically at the dendritic filopodia contact sites. Consequently, we generated a membrane-tethered calcium sensor, mem-GCaMP7s, which we imaged with high-resolution L-SIM2 and a frame rate of 15 fps. This setup allowed us to characterise calcium signalling specifically at filopodia contacts with an opposing neurite (Fig. 5A). We found several transients within the 60-s measuring period (Fig. 5B,C). Next, we examined whether Wnts are required for these transients. Using memNotum, we showed that membrane-tethered Wnt is necessary for generating calcium transients at the dendritic filopodia contact sites (Fig. 5D,E).

To investigate the requirement of WNT7A for local calcium signalling, we used a machine learning-based approach to identify dendritic filopodia and subsequently quantify the number of calcium transients in a 5 μm diameter circle around the dendritic filopodia contact site in our neuronal culture. In the control culture, we identified a specific signal of calcium transients at the filopodia tip and extracted the amplitude of the signals (Fig. 5F). Next, we overexpressed WNT7A (O.E. WNT7A) and found a significant increase in calcium transients (Fig. 5G), similar to iPSC-derived neurons exposed to recombinant WNT7A protein (Fig. 5H). Next, we blocked membrane-tethered Wnt signalling and investigated the effect on calcium transients at the membrane contact sites. We found that memNotum can block both overexpressed WNT7A and recombinant WNT7A (Fig. 5I-K), suggesting that WNT7A is necessary and sufficient for generating calcium transients at the dendritic filopodia contact sites. Furthermore, WNT7A can induce transients regardless of whether the signal is anchored to the membrane (through overexpression; Fig. 5J) or diffusible (as supplied as a recombinant protein in the media; Fig. 5K).

### WNT7A cytonemes induce precise local calcium signalling and control spine spacing

**Fig. 6.**
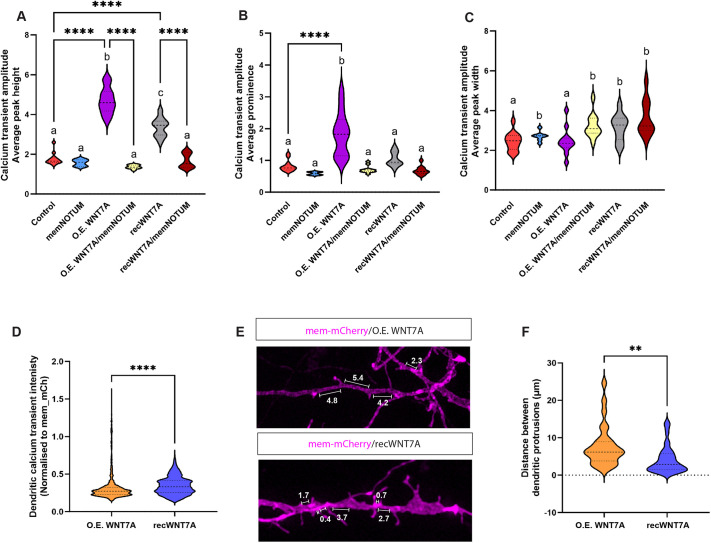
**Comparison of overexpressed and exogenously applied WNT7A in the induction of local calcium signalling and spine spacing.** (A) Quantification of calcium transient amplitude identified significant increases compared with control if neurons overexpressed WNT7A (O.E. WNT7A) or if the soluble protein was exogenously applied to the culture (recWNT7A). These increases were significantly attenuated when cells were co-transfected with memNotum. (B) A significant calcium amplitude peak prominence increase was observed in cells overexpressing WNT7A (O.E. WNT7A). (C) Calcium transient width was significantly altered in cultures expressing memNotum or after exogenous application of WNT7A (recWNT7A), compared to control or O.E. WNT7A groups. (D) Quantification of the dendritic calcium transient intensity identified a significant increase when comparing the overexpression of WNT7A to the exogenous application of recombinant WNT7A. (E) Representative images from iPSC-derived cortical neuron cultures either transfected with mem-mCherry and WNT7A or with mem-mCherry plus exogenous application of recombinant (rec.) WNT7A, followed by analysis of protrusion spacing along dendrites. (F) Quantification of the distance between dendritic protrusions identified a significant reduction in spacing when exogenously applying recombinant WNT7A, compared to overexpression of the protein. For statistical analysis of the calcium transient data, we performed direct comparisons within between groups using an unpaired, two-sided Student's *t*-test (**P<0.01, ****P<0.001), and used Tukey's Honestly Significant Difference (HSD) test with Holm–Bonferroni adjustments for multiple comparisons. We employed letter-based groupings for a compact representation of the significant differences amongst treatment groups. Groups not statistically different from one another share the same letter, while significantly different groups are designated distinct letters.

Next, we addressed the nature of the calcium transients to improve our understanding of the differences between focal WNT7A signalling on dendritic filopodia compared to a soluble WNT7A protein in the extracellular space. We found that the average peak amplitude is significantly increased by overexpression of WNT7A in the dendritic cytoneme generating neurons, whereas exposing the neurons to the recombinant protein led to a considerably smaller amplitude (Fig. 6A). Next, we measured how much the average peak would stand out from the surrounding signal baseline, which we defined as amplitude prominence. We found that the prominence was significantly increased between overexpressed, tethered WNT7A compared to the recombinant, soluble WNT7A (Fig. 6B). In support of these data, the peak width was significantly smaller in WT and WNT7A-expressing cells compared to the calcium peak after treatment with WNT7A protein (Fig. 6C). These data suggest that the nature of the calcium signal is spatially restricted when WNT7A is produced by the neuron and delivered by dendritic filopodia compared to a diffusible extracellular signal, leading to a broad and fuzzy calcium signal.

This blurring of the calcium transients at filopodia tips after exposure to secreted signal led us to the question of whether diffusible WNT7A leads additionally to the activation of ectopic calcium transients and, indeed, we found that treatment with diffusible WNT7A protein leads to ectopic activation of calcium transients along neurites, a finding not seen when the neurons produce endogenous or overexpressed WNT7A (Fig. 6D). Consequently, we tested whether the spacing between the dendritic filopodia is altered. Here, we observed that distances between the dendritic filopodia are significantly reduced after exposure to soluble WNT7A (Fig. 6E,F). In conclusion, these data suggest that, although soluble WNT7A can boost calcium signalling similar to WNT7A produced by the cells, the spatial information for precise calcium transients and, thus, the sites where the formation of dendritic filopodia contact sites is initiated become blurred.

### Membrane-tethered Wnts are required for synaptogenesis

**Fig. 7.**
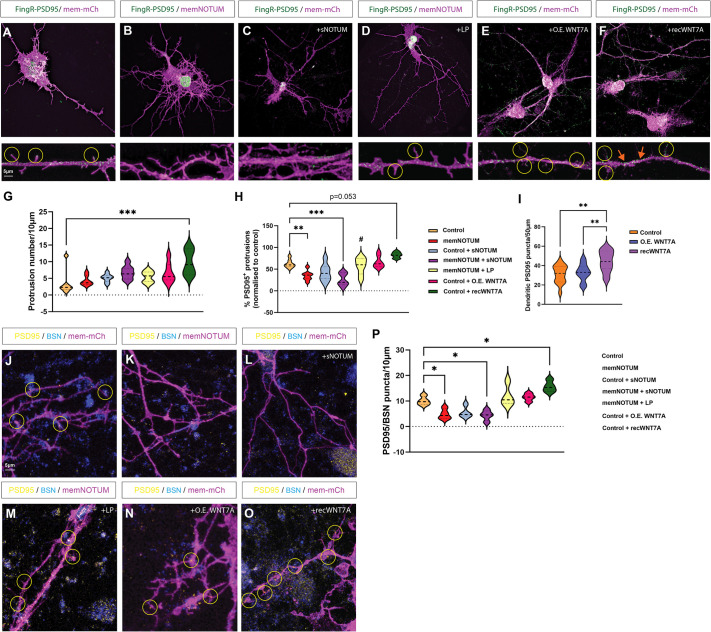
**Membrane-tethered Wnts are required for synaptogenesis.** (A-C) Cortical neurons were transfected with FingR-PSD95-GFP to tag PSD95 endogenously. Control neurons were co-transfected with mem-mCherry and showed strong PSD95 puncta localising to protrusions (A), which was lost when neurons were instead co-transfected with memNotum (B) or when control cultures were incubated with soluble, recombinant NOTUM (C). (D) Puncta expression could be rescued in memNotum transfected neurons by pre-treatment with the NOTUM inhibitor LP-922056. (E,F) Overexpression of WNT7A (O.E. WNT7A) did not alter PSD95 localisation to protrusions (E), whereas recombinant WNT7A (Rec. WNT7A) increased protrusion number (F). (G) Quantification of protrusion number in each group. (H) Quantification of PSD95-positive protrusions in each group. (I-O) Quantification (I) of ectopic PSD95 puncta on dendritic arborisation. Neurons were co-stained for PSD95 and bassoon (BSN) after transfection with either mem-mCherry (J), memNotum (K), mem-mCherry+soluble Notum (L), memNotum+LP-922056 (M), mem-mCherry+O.E. WNT7A (N), or mem-mCherry+Rec. WNT7A (O). (P) Quantification of PSD95/BSN puncta on transfected neurons. Statistical significance was addressed using one-way ANOVA with Dunnett's multiple comparison tests to compare relevant controls within groups and an unpaired, two-tailed Student's *t*-test to compare specific combinations. *P<0.05; **P<0.01; ***P<0.005. Experiments were performed in biological triplicate, with at least five fields per group analysed.

Next, we wanted to understand the requirement for spatially precise WNT7A signalling in the synaptogenic capacity of dendritic cytonemes. Therefore, we transfected iPSC-derived cortical neurons with the FingR construct, which tags endogenous levels of the postsynaptic scaffolding protein PSD95 with GFP (FingR-PSD95). Similar to WNT7A, we observed that FingR-PSD95 transfection of control neurons reveals PSD95 puncta localising to dendritic filopodia contact sites (Fig. 7A). To test for Wnt requirement, we inhibited the function of membrane-associated Wnts by memNOTUM and soluble NOTUM. We observed that the neurons co-transfected with FiNGR-PSD95 and memNotum display significantly reduced PSD95-positive protrusions (Fig. 7B,C,G,H). Treatment with the NOTUM inhibitor LP-922056 reversed the reduction of PSD95-positive protrusions in memNotum-transfected neurons (Fig. 7D,G,H). Next, we increased the WNT7A signal in the neuronal cultures. First, we performed a sequential transfection of the neurons, with mem-mCherry and untagged WNT7A to allow us to positively identify a neuronal subset with increased WNT7A protein, followed by FingR-PSD95-eGFP transfection. We observed that neurons overexpressing WNT7A do not increase the number of dendritic protrusions or PSD95-positive protrusions compared to WT neurons (Fig. 7E,G,H). However, if neurons are treated with soluble WNT7A protein, the neurites start to form ectopic protrusions, including PSD95-positive ones (Fig. 7F-H). Furthermore, we found a significant increase of PSD95 clusters at ectopic locations, e.g. along the neurite (Fig. 7F, orange arrows). These findings are in accordance with our previous results suggesting that WNT7A overexpression increases the signal amplitude and soluble WNT7A leads to ectopic calcium signalling sites (Fig. 6A-C).

Next, we wanted to address the differences between membrane-tethered and diffusible Wnts in relation to clustering of pre-synaptic markers because colocalisation of the postsynaptic compartment with the pre-synaptic active zone is required to confirm synapse formation. Therefore, we performed a similar experiment as outlined above, but post-stained cultures with antibodies against postsynaptic PSD95 and pre-synaptic BSN and performed confocal microscopy and subsequent quantification of the number of PSD95/BSN clusters localised at the protrusions of transfected neurons. We found a significant reduction in PSD95/BSN puncta when neurons were transfected with memNotum or treated with soluble NOTUM compared to the control neurons (Fig. 7J-L,P). This reduction could be reversed with treatment with the NOTUM inhibitor LP-922056 (Fig. 7M), confirming a requirement for Wnts to cluster PSD95 and BSN. Next, we overexpressed WNT7A in the neurons. We observed no significant change in the number of PSD95/BSN clusters, whereas there was a significant increase of PSD95/BSN colocalisation after treatment with diffusible WNT7A protein (Fig. 7O,P).

Based on these data, we conclude that WNT7A-carrying dendritic filopodia control the correct spacing of spines verified by colocalisation of pre-and postsynaptic markers at the contact sites. In contrast, diffusible WNT7A was also able to induce calcium transients; however, these were blurry (Fig. 6C). Consequently, the diffusible ligand led to PSD95/BSN clustering at ectopic sites, e.g. along neurites, leading to a disordered pattern of spines along the neurites.

## DISCUSSION

WNT7A signals through the Wnt/β-catenin pathway to regulate the formation and maturation of pre- and postsynaptic structures and is necessary for normal synaptic function (Teo and Salinas, 2021). For example, WNT7A/β-catenin signalling has been shown to promote spine formation and PSD95 expression in the murine hippocampus (Ramos-Fernández et al., 2019). WNT7A has also been shown to increase calcium entry to activate CaMKII in spines, leading to an increase in excitatory synapse formation in mice (Ciani et al., 2011). Although the beneficial role of WNT7A has been studied, it is still unclear how WNT7A is transported between the post- and presynaptic membrane to allow the establishment of a functional spine at a precise location.

We use super-resolution L-SIM2 to show that dendritic filopodia are decorated with WNT7A and transport the ligand to neighbouring neurons to cluster LRP6, thereby activating Wnt/β-catenin signalling. We show that WNT7A subsequently activates calcium transients and clustering of pre- and postsynaptic markers at the contact site of the dendritic filopodia, suggesting a requirement for membrane association of Wnts during synaptogenesis. In support of our hypothesis, data from *Drosophila* suggest that a membrane-tethered Wnt/Wg can function equally compared to a WT Wnt/Wg in wing development (Alexandre et al., 2014). Thus, our data indicate that lipophilic Wnt proteins are not freely diffusible but maintain membrane association. Long-range signalling might require a specific transport mechanism, such as an exovesicle-based transport or transport on long cytonemes (Zhang and Scholpp, 2019). We provide the first evidence that dendritic filopodia are WNT7A signalling cytonemes. In support of our finding, accumulating evidence suggests that these thin and highly polarised cytonemes can also operate in other contexts, such as during the transport of Wnt8a proteins in zebrafish gastrulation (Brunt et al., 2021; Stanganello et al., 2015), Wnt2b in the mouse intestinal crypt (Mattes et al., 2018), WNT3A in human embryonic stem cells (Junyent et al., 2020), Wnt3 in gastric cancer (Routledge et al., 2022), and Wnt5a in cancer-associated fibroblasts (Rogers et al., 2023) as well as in zebrafish development (Zhang et al., 2024).

Can we exclude that a meaningful proportion of the WNT7A protein is also freely diffusible? In our experiments, we find no evidence for the requirement of an additional pool of WNT7A other than the signals transported on dendritic filopodia. For example, we show that membrane-tethered WNT7A is similar in function to normal WNT7A, suggesting a continuous association with membranes during synaptogenesis. Furthermore, the inactivation of membrane-tethered Wnt proteins on the postsynaptic neuron cell is sufficient to inhibit the induction of calcium transients and synaptic protein clustering. Moreover, WT neurons (which also produce WNT7A) in the same cell culture dish cannot rescue the lack of membrane-bound WNT7A on dendritic filopodia tips needed for synapse formation, arguing against a pool of freely diffusible WNT7A or ligand-loaded exovesicles. Finally, we also provide evidence that, in contrast to endogenously produced WNT7A, the addition of soluble WNT7A changes the formation of spine patterning by inducing additional connections in abnormal locations with ectopic sites for calcium transients and pre- and postsynaptic clusters, which could disrupt the normal flow of information within neural circuits leading to altered signal processing and miscommunication between neurons.

In conclusion, our work bridges a fundamental knowledge gap by explaining how and where WNT7A functions in excitatory synapse formation. Our data further challenge the long-standing concept of separating the chemical signalling events (by Wnts and calcium) from the morphological structure of the dendritic filopodia in synaptogenesis. We show that dendritic cytonemes are necessary for mobilising WNT7A and trafficking the ligand to dendrites of neighbouring neurons. WNT7A on the tips of dendritic cytonemes then triggers the local induction of calcium signalling, leading to the clustering of pre-and postsynaptic markers and paving the way to the formation of new, functional synapses at the correct location for properly functioning neuronal network formation to occur.

## MATERIALS AND METHODS

### Plasmids and antibodies

The following plasmids were used in transfections: pCS2+-membrane-mCherry (Mattes et al., 2018), pCAG-mGFP membrane-bound GFP (Addgene #14757), pcDNA3.1-WNT7A-GFP, pcDNA3.2-WNT7A-V5 (Addgene #43816), 7×TRE-SuperTOPFlash-NLS-mCherry (Moro et al., 2012), pCS2+-LifeAct-GFP, pCAG-PSD95.FingR-eGFP-CCR5TC (Addgene #46295), Lck-mScarlet-I (Addgene #98821), pCS2+-Vhh-CD8-mCherry [cloned from Gal4/LexA-Vhh-CD8-mCherry (M. Affolter, University of Basel, Switzerland) into pCS2+ vector using XhoI/XbaI], pCS2+-GAP43-jGCaMP7 s [cloned from pGP-CMV-jGCaMP7 s (Addgene #104463) into pCS2+-GAP43-GFP using XbaI/SnaBI], pCS2+LRP6-eGFP (gift from G. Davidson, KIT, Karlsruhe, Germany), GFP-Bsn 95-3938 (gift from Eckart Gundelfinger, Leibniz Institute for Neurobiology, Magdeburg, Germany), pcDNA3.1-Notum-CD8-mCh [assembled by Gibson cloning with pcDNA3.1-hNotumFL (gift from J. P. Vincent, Francis Crick Institute, London, UK) and pCS2+-Vhh-CD8-mCherry], PSD-95-pTagRFP (Addgene #52671).

The following primary antibodies were used for immunofluorescence: anti-WNT7A (abcam, ab100792), anti-LRP6 (BioTechne, FAB1505R), anti-bassoon (abcam, ab82958), anti-PSD95 (abcam, ab18258), anti-PSD95 (abcam, ab13552), anti-flotillin 2 (Santa Cruz Biotechnology, sc-28320), anti-flotillin 2 (abcam, ab113661), anti-myosin-X (C-1) (Santa Cruz Biotechnology, sc-166720), anti-Evi (EMD Millipore, YJ5). The following Alexa Fluor-conjugated (Thermo Fisher Scientific) secondary antibodies were used for immunofluorescence: goat anti-rabbit 488 (abcam, ab150077), goat anti-mouse 647 (abcam, ab150115), donkey anti-mouse 488 (abcam, ab150105), donkey anti-goat 647 (abcam, ab150135).

### Cell culture

#### iPSC maintenance

iPSCs (GM23280A, KOLF2.1J) were obtained from the Coriell Institute for Medical Research and the iNDI consortium (gift from Prof W. Skarnes, The Jackson Laboratory), respectively. Lines were maintained as colonies on human ES-qualified Matrigel (Corning) in StemFlex (StemCell Technologies). Colonies were routinely passaged in a 1:6 split using EDTA and banked. All cell lines were tested regularly for *Mycoplasma* by endpoint PCR testing every 3 months and broth tests every 12 months.

### Differentiation of iPSCs into cortical neurons

Neurons were derived as previously described (Strano et al., 2020). Briefly, iPSCs were plated as colonies onto Matrigel and differentiated by treatment with neuronal differentiation media (DMEM/F12:Neurobasal in a 1:1 ratio, HEPES 10 mM, N2 supplement 1%, B27 supplement 1%, Glutamax 1%, ascorbic acid 5 μM, insulin 20 μg/ml) supplemented with SB431542 (10 μM) and LDN8312 (0.2 μM) from days (D) 0-D12, replacing media daily. On D12, cultures were re-plated using Accutase in neuronal differentiation media supplemented with basic fibroblast growth factor (bFGF; 20 ng/ml), CHIR-99021 (1 μM), and Y-27632 (50 μM). On D13-D18, media was changed daily [neuronal differentiation media supplemented with bFGF (20 ng/ml), CHIR-99021 (1 μM)]. Cells were either banked as neural progenitor cells, stained for cortical neuron lineage markers, or maintained in culture until D60-D80 for experimentation. Cells were maintained in differentiation media supplemented with L-ascorbic acid (200 μM), BNDF (20 ng/ml), GDNF (10 ng/ml), and the small-molecule NOTCH inhibitor Compound E (0.1 μM) for 7 days before Compound E was removed. Cultures were replenished every 3-4 days with a 50:50 media change. Treatments with 1 μg/ml of recombinant human NOTUM protein (Bio-Techne) or 100 nM LP-922056 (kind gift from Alzheimer's Research UK Drug Discovery Alliance, London, UK) were performed for 48 h prior to image acquisition.

### SH-SY5Y neuroblastoma neuronal differentiation

SH-SY5Y neuroblastoma cells were obtained from Akshay Bhinge (LSI Exeter, UK) and regularly tested for *Mycoplasma* by endpoint PCR testing every 3 months and broth tests every 12 months. SH-SY5Y were plated onto PDL and laminin-coated 6-well plates in a starving medium (DMEM/F12 plus, 1% fetal bovine serum, and 10 μM retinoic acid). From D0 to D8, media was replaced every other day. Cells were then cultured in neuronal differentiation media (DMEM/F12:Neurobasal, 10 mM HEPES, 1% N2 supplement, 1% B27 supplement, and 1× Glutamax) supplemented with retinoic acid (10 μM) and BDNF (10 ng/ml) with media changes every other day until D40-60 when cells were plated for experimentation.

### Neuronal transfection

Unless otherwise stated, neuronal cultures were transfected with equimolar ratios of plasmid DNA totalling 1.5 μg using a calcium-phosphate method as previously described (Hawkins et al., 2022). A plasmid/CaCl_2_ (12.4 mM) master mix was prepared for each combination to be transfected in Hank's balanced salt solution (HBSS), mixing gradually (1/8 of DNA:CaCl_2_ mixture/addition) to prevent the formation of large transfection complexes. Complexes were incubated for 20 min at room temperature, then added to cultures (no more than 1/10 of total volume) and incubated at 37°C for 4.5 h before a sodium acetate (300 mM in DMEM: F12) washed at 37°C for 5 min to dissolve precipitated complexes. Cultures were maintained in neuronal differentiation media supplemented for 24-48 h prior to imaging analyses.

### AGS cell culture and transfection

The primary gastric adenocarcinoma cell line AGS was a kind gift from Dr Toby Phesse (Cardiff University, UK). AGS cells were maintained in RPMI-1640 (Sigma-Aldrich), supplemented with 10% fetal bovine serum. In addition, cells were routinely passaged with TrypLE (Thermo Fisher Scientific). Transient transfections of AGS cells were performed using FuGeneHD (Promega) according to the manufacturer's protocol (using a 3:1 FuGene:DNA ratio).

### SuperTOPFlash-based Wnt reporter assay

To determine the functionality of the membrane-tethered NOTUM construct, 5×105 AGS cells were reverse-transfected with SuperTOPFlash reporter plasmid (7×TRE-NLS-mCherry), and 5×10^5^ AGS cells reverse-transfected with the indicated plasmids in 6-well plates. After 24-h incubation, both cell types were trypsinised and counted, and 5×10^5^ of each population were co-cultured in 6-well plates for a further 48 h before fixation and post-staining with DAPI for total nuclei quantification. Image acquisition was performed on a Leica DMI6000 SD microscope using a 20× objective. For all assays, at least four images were taken in random locations for three biological repeats. The fluorescence intensity of mCherry-positive nuclei was measured using Fiji software. The number of DAPI-positive nuclei was also counted as a measure of proliferation. To assay the activity of recombinant WNT7A and overexpression of WNT7A, 5×10^5^ AGS cells were reverse-transfected with the SuperTOPFlash reporter with or without untagged WNT7A plasmid (Addgene #43816), WNT7A-GFP plasmid, or 50 ng/ml recombinant human WNT7A protein (abcam, ab129138) and quantification of positive nuclei performed after 48 h, as described above.

### Antibody staining and image acquisition

Cells were plated onto 1.5H precision coverslips (Zeiss), and following indicated treatment/incubation, cells were immediately fixed using modified MEM-Fix (4% formaldehyde, 0.25-0.5% glutaraldehyde, 0.1 M Sorenson's phosphate buffer, pH7.4) (Bodeen et al., 2017; Rogers and Scholpp, 2020) for 7 min at 4°C. Aldehydes were subsequently quenched by incubation with NaBH_4_ (0.1% w/v) for 7 min at room temperature. Further quenching was performed by three 10 min washes in PBS-glycine (0.2 M). Cells were then incubated in permeabilisation solution (0.1% Triton X-100, 5% normal goat serum, 0.1 M glycine in 1×PBS) for 1 h at room temperature. Primary antibodies were diluted in incubation buffer (0.1% Tween20, 5% normal goat serum in 1×PBS) and coverslips mounted on 40 µl spots overnight at 4°C in a humid environment. Coverslips were then washed with 1×PBS three times for 5 min each wash before mounting on 40 µl spots of secondary antibodies with or without Phalloidin-405 (for actin cytoskeleton experiments) diluted in incubation buffer for 45 min at room temperature. Coverslips were then washed three times for 5 min each wash with 1×PBS before mounting onto glass slides using ProLong Diamond Antifade mountant (Invitrogen) and left to dry for 24 h at 4°C before imaging. Confocal microscopy was performed on an inverted Leica TCS SP8 X laser-scanning microscope using the 63× water objective. SIM for transfection studies and immunofluorescent antibody staining were performed on the Zeiss Elyra7 Lattice SIM-squared system using 40× and 63× objectives, employing Apotome and L-SIM2 modes, respectively.

### Calcium imaging

Live calcium imaging was performed on the Elyra7 Lattice SIM-squared system in laser widefield, HILO mode for basic calcium transient analysis. For constant calcium imaging studies, a Zeiss LSM880 confocal microscope equipped with an Airyscan detector, employing a 63×/1.46 NA oil immersion objective, was utilised. Membrane-tethered GCaMP7S (mem-GCaMP7S) was excited using a 488 nm wavelength to visualise calcium transients. The Airyscan detector was consistently operated in ‘Fast mode’. *z*-stacks were captured to fully represent the filopodial volume, ranging from 3 to 5 μm with a 0.2 μm *z*-interval. Time-lapse recordings were set at intervals from 200 ms, contingent on the expected dynamics of the calcium events. Image acquisition parameters, such as pixel dwell time and resolution, were optimised to ensure the most suitable balance between speed and resolution.

### Calcium signalling analysis

Post-acquisition, data underwent Airyscan processing in Zeiss Zen (v.3.2) software suite to enhance resolution and signal-to-noise ratio. The data were imported into Imaris version 10 for quantification of calcium transients. Primarily, background subtraction and normalisation of the membrane maker were conducted. Then, within Imaris, using the advanced filament tracer module set on the membrane channel, a thresholded machine-based learning algorithm was deployed to accurately identify dendrites, filopodia, and spines. Following this identification, calcium data from single filopodia were extracted as a function of time (300 frames from 20 min of data capture) using the Imaris track module. The data extraction specifically targeted the tips of these structures, and the average fluorescence intensity was extracted.

### Calcium data statistical analysis

For the analysis of time-dependent trends, data were specifically curated from images wherein filopodia and spines were in close proximity to adjacent transfected dendrites that also displayed filopodia and spines. After selection, the data were subjected to normalisation to ensure uniformity and comparability. All statistical analyses were conducted using Python, with the primary libraries being pandas for data manipulation, statsmodels for statistical modelling, and seaborn for data visualisation. To evaluate the impact of time, treatment, and their potential interaction on fluorescence intensity mean (FIM), we employed a Generalized Linear Model (GLM) with the formula: FIM∼Time+Treatment+Time×Treatment. The Gaussian family was chosen for the GLM due to the continuous nature of the FIM response variable. The resultant model provided coefficients, standard errors, and *P*-values for each term in the model. Following the GLM, a post-hoc analysis was conducted to discern pairwise differences between treatment levels. We applied Tukey's Honestly Significant Difference (HSD) test, which offers pairwise comparisons for all treatment levels. To account for the problem of multiple comparisons, *P*-values from Tukey's HSD test were further adjusted using the Holm–Bonferroni method. We constructed letter-based groupings to provide a compact representation of the significant differences amongst treatment groups. Groups not statistically different from one another share the same letter, while significantly different groups are designated distinct letters. Average FIM values were plotted with their standard errors as a function of time for each treatment group. FIM were also subjected to peak analysis using the Python SciPy library. Peaks in the FIM data were identified based on specific criteria for peak properties, such as height/amplitude, distance between peaks, prominence, width at half-height, and threshold for peak prominence. The criteria for peak detection were as follows: minimum peak height (amplitude): 0.1; minimum distance between peaks: 5 frames; minimum peak prominence: 0.05; and width threshold: 0.5-20 frames. The chosen criteria ensured robust peak detection while minimising the inclusion of noise or minor fluctuations in the data. To evaluate the effects of the different treatments (treat) and filopodia/spine IDs (Filo) on the detected peak properties, a two-way analysis of variance (ANOVA) without interaction was conducted. This was followed by a one-way ANOVA specifically focusing on the effect of treatments on peak count, average peak height, average peak prominence (an indicator of how distinct and prominent peaks are compared to their surroundings), and average peak width. Post-hoc pairwise comparisons were performed using Tukey's HSD test to identify specific treatment pairs with significant differences in their peak properties.

### Quantifications and statistical analyses

All protrusion quantifications were calculated from *z*-stack images of cells stained with phalloidin or expressing membrane-mCherry or lck-mScarlet-I using Imaris software (Oxford Instruments). All experiments/conditions were repeated in biological triplicate and at least 400 μm of dendritic arborisation was analysed/experiment. For dendritic spine quantification, filopodia were defined as long, thin protrusions without a clear spine head, characterised by a length greater than twice the width of the spine. Stubby spines were identified as short, thick protrusions with a length approximately equal to their width and lacking a distinct head, and mushroom spines were defined as protrusions with a large, bulbous head and a thin neck, where the head width was at least twice the width of the neck. Each type was quantified on a minimum of 400 μm of dendritic arborisation across three randomly selected fields for each experiment. Quantification was performed using Imaris software, enabling the recording of further parameters, including protrusion density and length. Protrusion length was measured from the tip of the filopodia to the base, where it contacted the neurite. In the case of branching protrusions, one branch (the longest) would be measured. Quantification of the apposition (co-clustering) or colocalisation of Wnt signalling components (WNT7A and LRP6) and synaptic markers (bassoon and PSD-95) detailed in Figs 1, 2, and 7 was performed manually using the same parameters. Colocalisation analyses of cytoneme markers, morphotrap, and memNotum experiments were performed using the ‘Coloc’ function in Imaris using a protrusion tip threshold and normalised to a 10 μm length of the neurite. Significance was tested using an unpaired, one-tailed Student's *t*-test (parametric) against the relative controls. One-way or two-way ANOVA with Dunnett's or Bonferroni post-hoc tests were performed for multiple comparisons.

## Supplementary Material

10.1242/develop.202868_sup1 Supplementary information
