# Supplementary material for: WNT7A-positive dendritic cytonemes control synaptogenesis in cortical neurons
Source: Development. 2024 Dec 9;151(23):dev202868. doi: 10.1242/dev.202868 (PMC11658684; doi:10.1242/dev.202868)
Supplement: Supplementary information [file develop-151-202868-s1.pdf]

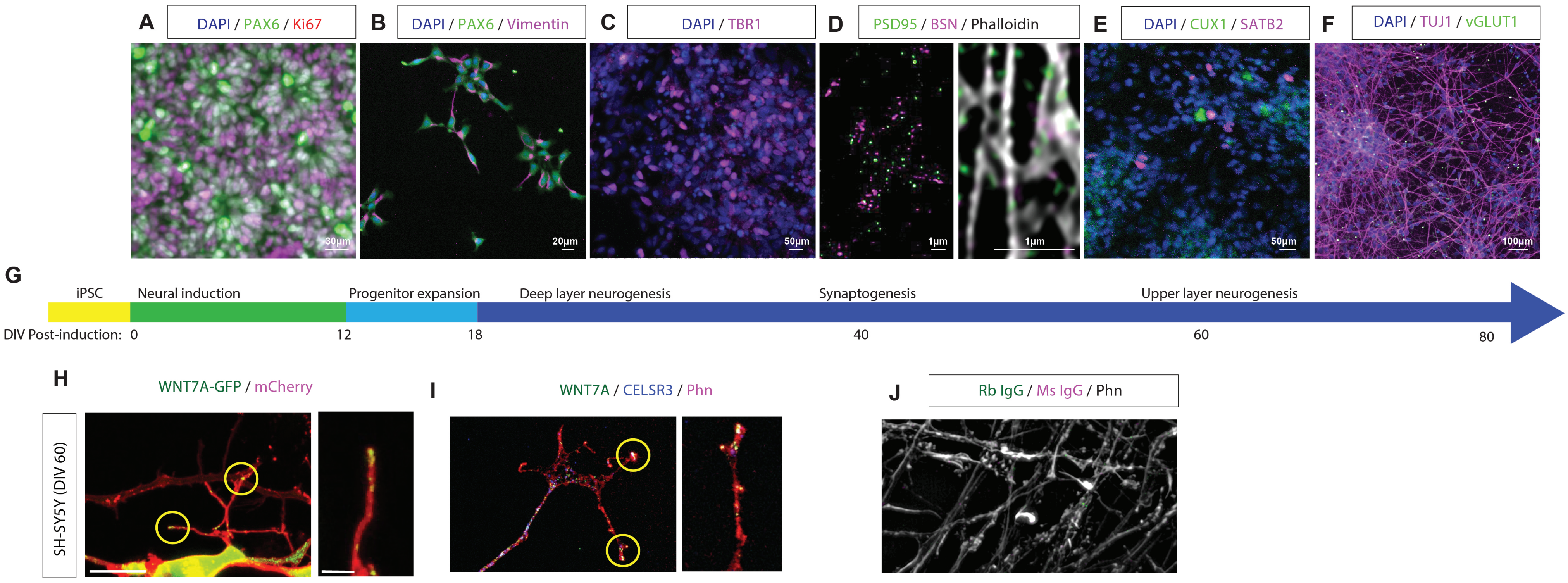

Fig. S1. related to Figure 1. Quantification methodology and negative controls. Characterisation of iPSC differentiation into cortical neurons from PAX6/Vimentin positive progenitors (**A**/**B**) to deep layer cortical neurogenesis (TBR1+; **C**), early synaptogenesis (**D**), upper layer neurogenesis (Cux1/SATB2+; **E**), leading to complex neuronal networks (**F**). (**G**) Schematic timeline of iPSC-derived cortical neuron differentiation. (**H**) Overexpression of Wnt7a-GFP in SH- SY5Y-derived neurons shows localisation to the dendritic filopodia tips (yellow circles). (I) Antibody staining for Wnt7a protein in SH-SY5Y-derived neurons indicates co-localisation with CELSR3 the the filopodia tips (yellow circle). (**J**) IgG isotype controls for primary antibodies.

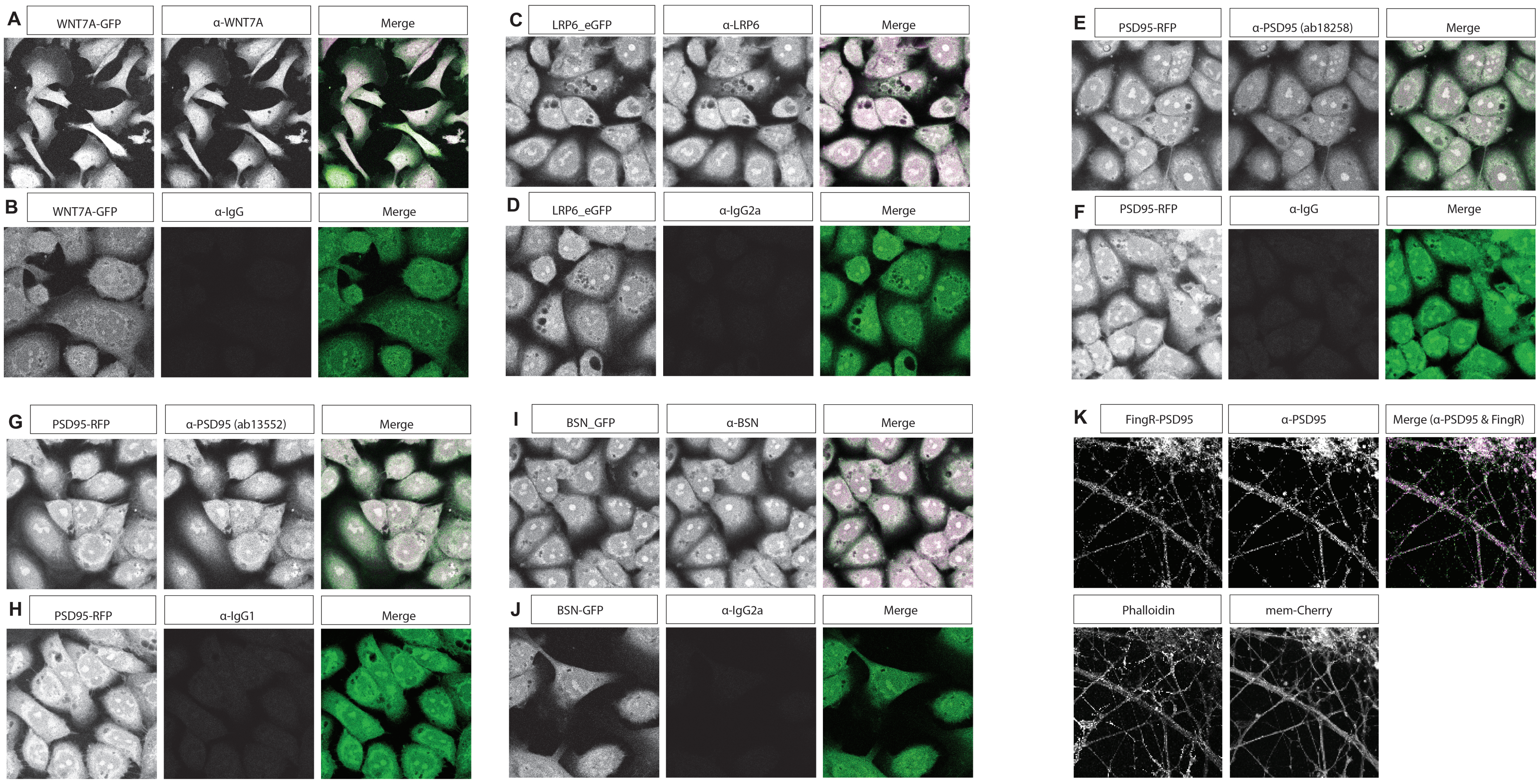

Fig. S2. Antibody specificity controls. To confirm the antibodies used to positively identify Wnt signalling components, and synaptic markers were specific we performed a number of AGS transfections followed by post-staining with antibodies and isotype controls. Specifically, cells were transfected with Wnt7a-GFP and subsequently stained with α-Wnt7a or α- IgG antibodies (**A, B**), LRP6-eGFP followed by staining with α-LRP6 or α-IgG2a antibodies (**C, D**), PSD95-RFP and stained with α-PSD95 (ab18258), α-IgG, α-PSD95 (ab13552), or α-IgG1 antibodies (**E, F, G, H**), and GFP-BSN followed by staining with α-BSN or α-IgG2a antibodies (**I, J**). To confirm endogenous tagging of PSD-95 with the FingR construct was specific, SH-SY5Y neurons were transfected with FingR-PSD95_eGFP and mem-mCherry and post-stained with α-PSD95 (ab18258) and Phalloidin (K).

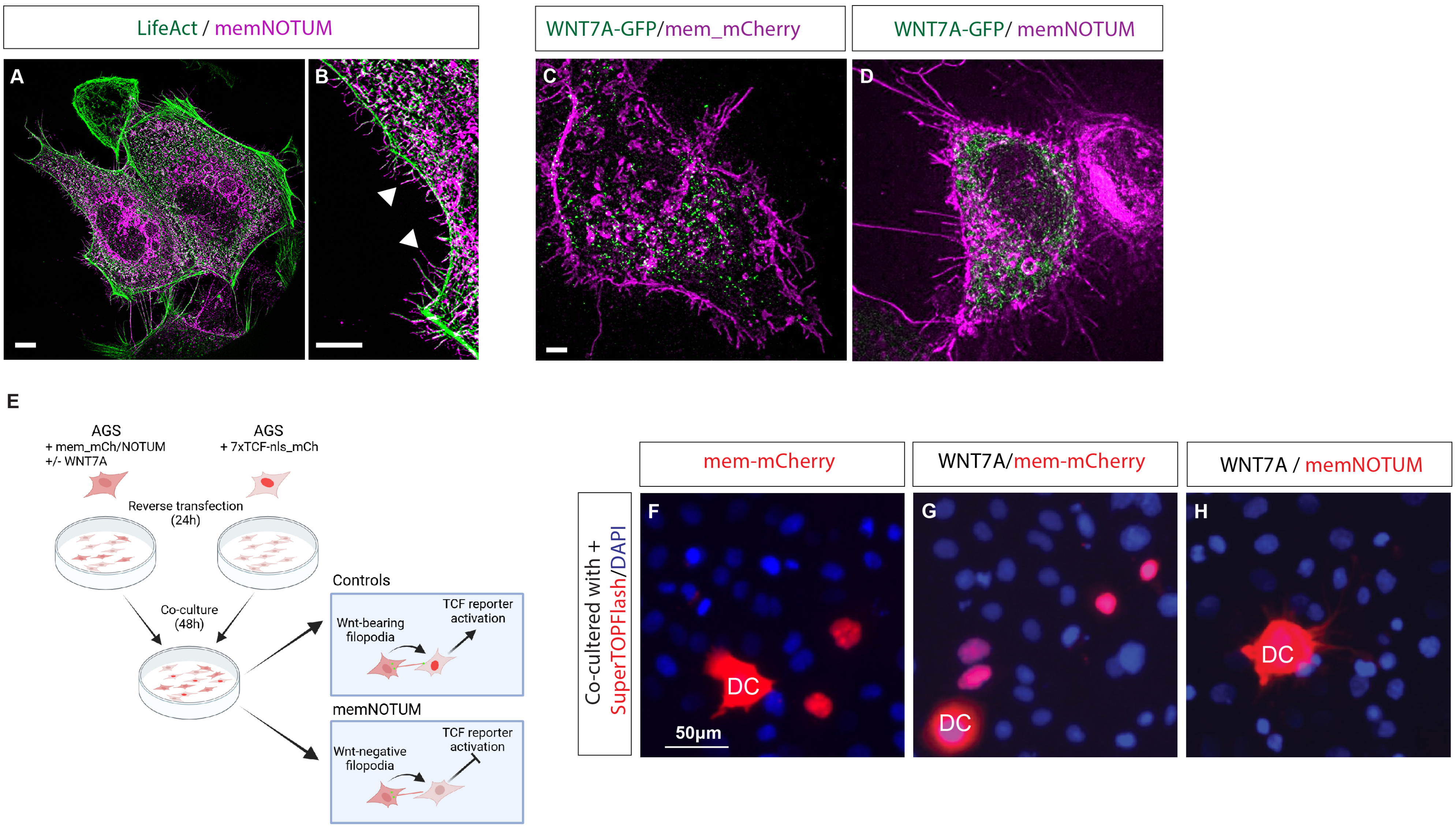

Fig. S3. Generation and characterisation of membrane-tethered Wnt 'scissor.' (**A**) Transfection of human AGS cells with memNotum shows strong expression on all cell membranes and cellular filopodia (**B**). Cells were co-transfected with LifeAct-GFP for visualisation of the actin cytoskeleton. (**C**) Co-transfection of the membrane marker, mem-mCherry, and Wnt7a- GFP show localisation of Wnt7a-GFP to filopodia tips. (**D**) Co-transfection of memNotum and Wnt- 7a-GFP reduces Wnt7a-GFP positive filopodia. (**E**) Schematic of SuperTOPFlash-based TCFx7- NLS-mCherry reporter assay set-up. (**F**) TCF reporter expression can be observed in cells around the control donor cell (DC) population (mem-Ch). (**G**) Reporter activity is increased if the donor cell population is co-transfected with Wnt7a-GFP (mem-mCherry). (**H**) TCF reporter activity is significantly reduced if donor cells are transfected with memNotum.

Development • Supplementary information
